# Editorial: Body composition and cardiovascular health

**DOI:** 10.3389/fnut.2024.1414103

**Published:** 2024-05-13

**Authors:** Ian G. Davies, David Clayton, Richard Kirwan, Michael A. Schmidt

**Affiliations:** ^1^Research Institute of Sport and Exercise Science, Liverpool John Moores University, Liverpool, United Kingdom; ^2^School of Science and Technology, Nottingham Trent University, Nottingham, United Kingdom; ^3^Sovaris Aerospace, Boulder, CO, United States

**Keywords:** body composition, cardiovascular disease, muscle mass and fat mass, sarcopenia, vitamin D

Cardiovascular disease (CVD) displays a major global concern, has a strong relationship with obesity and an emerging association with lower levels of muscle mass or sarcopenia, highlighting the importance of measuring body composition in cardiovascular (CV) health research (1, 2). Several methods are available to measure body composition, including gold standards such as Dual-Energy X-ray Absorptiometry (DXA) and Magnetic Resonance Imaging (MRI), and other more accessible methods, such as Bioimpedance (BIA) (3). These measures provide analysis of skeletal muscle and body fat metrics, that influence basal metabolic rate (BMR) and metabolic and CV health. In the absence of sophisticated equipment, and especially for large-scale observational studies, Body Mass Index (BMI) and waist circumference (WC) are useful in CVD prediction models either as an exposure or covariate but novel indices such as the Body Roundness Index (BRI) are also gaining traction. BRI is calculated from height and WC and is proposed to improve determination of total body fat percentage and visceral adipose tissue mass. However, it remains equivalent in predicting CVD related disease (4), highlighting the need for further research.

Gaining a deeper understanding of body composition and its related measures, provides a greater insight of CVD risk and will drive future research innovations, which may lead to preventative strategies. This Research Topic explores the role of body composition in cardiovascular health, with four novel studies investigating cardiovascular endpoints; risk factors such as blood pressure and thrombotic risk; and the relationship of vitamin D with CVD risk.

A study by Zhang et al. focused on the relationship of BRI with composite cardiovascular endpoints (CCE), consisting of stroke, myocardial infarction, and CVD death, conducted in rural regions of China (*n* = 13,209). This is an understudied area where the population has a different diet and lifestyle compared to urban areas of China, and there is limited longitudinal research on BRI and CV health. The results revealed both moderate and high BRI trajectories (over a 6-year period) increased CCE risk. While this evidence highlights the potential utility of BRI as a predictive tool, other studies have shown varied results. For example, analysis of the US National Health and Nutrition Examination Survey (NHANES) database (*n* = 47,356) over approximately 8 years, found the relationship with BRI and all-cause and cardiovascular mortality was U-shaped (5). This suggests that both low and high BRI values are associated with increased (cardiovascular) mortality risk, while moderate values are associated with lower risk. Lower BRI values, are linked with malnutrition and muscle atrophy (5) emphasizing the significance of measuring key metrics of sarcopenia, such as Hand Grip Strength (HGS) and/or muscle mass. However, there is a lack of longitudinal evidence using muscle mass, along with conflicting cross-sectional data, regarding cardiovascular health.

In another of our Research Topic studies, Bu conducted a longitudinal analysis of middle-aged Korean adults (*n* = 2,669), showing an inverse association between relative skeletal muscle mass (RSM) (as measured by BIA) and incident hypertension over a 16-year follow-up period. The participants were categorized into tertiles (T) of RSM, revealing higher hypertension risk in T1 and T2 compared to T3 (the highest RSM). Notably, a recent meta-analysis demonstrated that sarcopenia is associated with a 1.67 and 1.31-fold increased risk of stroke and cardiovascular disease (CVD), respectively (6), highlighting the potential significance of hypertension and muscle health in CVD.

Mechanistic evidence supports vitamin D's involvement in various metabolic pathways relevant to skeletal muscle atrophy and vascular function respectively (7, 8), which may present a bridge between body composition, vitamin D status, and CVD. A study by Che et al. in our Research Topic, analyzing the US NHANES database (*n* = 17,467), revealed a non-linear (U-shaped curve) inverse association between vitamin D levels and elevated blood pressure, even after adjusting for BMI in individuals without prior hypertension diagnosis. While epidemiological studies suggest increasing doses of vitamin D have an inverse association with hypertension (9), evidence is uncertain from randomized controlled trials (RCTs) (10). The Che et al. study suggests an upper ceiling of vitamin D intake, which is consistent with other research when high plasma levels may have a detrimental effect in other areas such as fracture risk (11). There is emerging evidence on the relationship with skeletal muscle, hypertension, and vitamin D, implicating the role of mitogen-activated protein kinase signaling and angiotensin (12), but large-scale dose response and mechanistic studies are needed for a more comprehensive understanding.

Basal metabolic rate (BMR), which is heavily influenced by body composition, is notably higher in obesity, but primarily driven by fat-free mass (13). Recently, it has emerged as a novel factor impacting CVD risk. In our Research Topic, employing Mendelian randomization (MR) methods, Huang and Xie investigated the novel causal relationship between BMR and venous thromboembolism (VTE) in European populations. Their findings uncovered a significant positive association between elevated BMR levels and heightened VTE risk, encompassing pulmonary embolism (PE) and lower extremity deep vein thrombosis (DVT). Potential mechanisms include increased reactive oxygen species (ROS), a pro-inflammatory state, and enhanced platelet activation and aggregation, fostering a prothrombotic milieu. Obesity alone, and its coexistence with low muscle mass and strength (sarcopenic obesity), correlates with VTE and related outcomes (14, 15), influencing BMR in a nuanced manner. While lower muscle mass reduces BMR, when coupled with excess adiposity (particularly visceral adipose tissue), a prothrombotic environment may persist. The study in our Research Topic sets the stage for further MR research into VTE and body composition, emphasizing the need to address the nuanced interplay of body composition factors.

In summary, while the results of the above studies showcase novel evidence regarding body composition and CV health, there is clearly more research needed in this area. The Research Topic offers the latest evidence on how novel indices of body composition, skeletal muscle mass, BMR and vitamin D impact health, from cardiovascular risk factors through to cardiovascular endpoint and mortality. The hope is the reader will be engaged not only by the evidence presented but will also be intrigued by how the role of body composition in CV health is nuanced, requiring further research that will potentially lead to preventative strategies.

## Author contributions

ID: Writing – original draft, Writing – review & editing, Conceptualization. DC: Writing – review & editing, Conceptualization. RK: Writing – review & editing, Conceptualization. MS: Writing – review & editing, Conceptualization.
